# Rapid neurological recovery with spontaneous resolution of acute subdural hematoma after severe head trauma: A case report of auto-decompression phenomena

**DOI:** 10.1016/j.ijscr.2025.110973

**Published:** 2025-01-25

**Authors:** Barnabas Obeng-Gyasi, Anoop S. Chinthala, Alexei Christodoulides, Josue Ordaz, Gordon Mao

**Affiliations:** Department of Neurological Surgery, Indiana University School of Medicine, Indianapolis, IN, USA

**Keywords:** Traumatic brain injury (TBI), Acute subdural hematoma (aSDH), Spontaneous resolution, Coagulopathy, Neurological recovery, Case Report

## Abstract

**Introduction:**

The spontaneous resolution of acute subdural hematoma (aSDH) represents an ill-defined but clinically significant phenomenon in traumatic brain injury (TBI). While surgical evacuation remains the standard of care for significant aSDH, rare cases of spontaneous resolution, termed auto-decompression in literature, suggest alternative pathways of hematoma clearance that warrant further investigation.

**Case presentation:**

We discuss the case of a 40-year-old male with background seizure disorder who fell off a ladder. His Glasgow Coma Score (GCS) at presentation was 5. Brain Computed Tomography (CT) scan revealed bilateral aSDH and multiple skull fractures. Within 24 h, his GCS quickly improved to 9 T. Repeat brain CT done 72 h post-trauma showed resolution of the aSDH following non-operative management.

**Discussion:**

Spontaneous resolution of bilateral aSDH with rapid neurological improvement is a rare but possible occurrence, often attributed to auto-decompression phenomenon in patients with severe head trauma and specific predisposing factors. Our discussion revolves around this patients presentation with polytrauma, complex skull fractures, history of craniotomy, and acute coagulopathy contributing to the spontaneous resolution of the hematoma. Given the complex nature of TBI and the unpredictable course of recovery, clinicians must remain vigilant in continuously reassessing neurological status.

**Conclusion:**

This case discusses the unpredictable nature of TBI and highlights the rapid and unexpected resolution of aSDH in a patient with complex neurosurgical history, coagulopathy, and polytrauma. The findings showcase the problems of polytraumatized patients and exemplify the importance of individualized care even when initial signs indicate poor prognosis.

## Highlights

• Rare case of spontaneous hematoma resolution post-TBI
• Extensive skull fractures, coagulopathy, and chronic alcoholism are factors that can aid in spontaneous resolution
• Emphasized the unpredictable recovery trajectory in severe TBI management

## 1 Introduction

Traumatic Brain Injury (TBI) is a leading cause of mortality and long-term disability worldwide, with an estimated 69 million individuals sustaining such injury annually [1]. In 2013, the United States recorded around 2.5 million visits to emergency departments, 282,000 hospital admissions, and 56,000 fatalities associated with traumatic brain injury [2]. The severity of TBI can range from mild to severe, with the latter often associated with devastating neurological deficits and poor prognosis. Fall from ladders is a common cause of TBI, contributing to a significant proportion of these injuries, especially among young adults [[3], [4], [5]].

The clinical outcome of individuals with severe TBI is highly unpredictable [6,7]. In some cases, despite poor clinical presentations, patients may experience remarkable neurological recovery. This recovery process is influenced by factors such as age, the immediacy and nature of medical and surgical interventions, as well as the intensity of rehabilitation services [[8], [9], [10], [11]]. Notably, there have been rare instances where patients have demonstrated spontaneous resolution of bilateral subdural hematomas post-trauma, referred to as auto-decompression, adding to the complexity and unpredictability of TBI outcomes [12,13].

This case highlights the importance of considering a patient's previous neurological history, unique skull and brain pathology, and the role of coagulopathy in TBI management. We discuss the potential rare occurrence of auto-decompression phenomena, the general elusive nature of prognostics in TBI, and the challenges inherent in predicting recovery trajectories.

## 2 Methods

This work was completed in line with the SCARE criteria [14].

## 3 Case presentation

A 40-year-old male was brought to the emergency department following a fall from a 12 ft. ladder. He had previous craniotomy for trauma 6 years back and background seizure disorder that was well controlled with Levetiracetam 500 mg daily. He had a significant history of alcohol use. While he had a known seizure disorder managed with Levetiracetam 500 mg daily, no seizure was documented at the time of injury. Due to questionable medication compliance, it remained unclear whether the fall was accidental or precipitated by a seizure.

According to emergency medical personnel, the patient was combative on-scene, with a GCS of 5. Before arriving in the emergency department, he was intubated and sedated for airway protection. Noteworthy examination findings included active epistaxis and a rapidly evolving left periorbital hematoma. Upon admission, he had a GCS score of 3 T and was hypotensive, with a systolic blood pressure in the 60s and a diastolic blood pressure in the 40s. Initial resuscitation included transfusions of packed RBCs and IV crystalloid, but the patient remained hypotensive requiring further stabilization with four vasopressor agents. FAST exam was negative. CT abdomen and pelvis did not reveal active hemorrhage. The trauma team identified a moderate hemothorax, which led to the insertion of a left chest tube with 300 mL output. A total of 4 units of fresh frozen plasma, 4 units of packed RBCs and 2 L of crystalloid was transfused to maintain hemodynamic stability. CT imaging further revealed left rib fractures 1–9, right rib fractures 1–8, thoracic spine fracture T6-T10, transverse process fractures T5-T9, left distal clavicle fracture, and bilateral scapulae fractures, prompting spinal precautions, including application of a cervical collar and a Thoracolumbosacral Orthosis (TLSO) brace. He was also placed on levetiracetam given the fact seizure was a concern. Toxicology screen was negative and blood alcohol level was <10 mg/dL. Other notable injury found on imaging was a grade 5 splenic injury with splenic pseudoaneurysms and left adrenal hematoma.

**Fig. 1:**
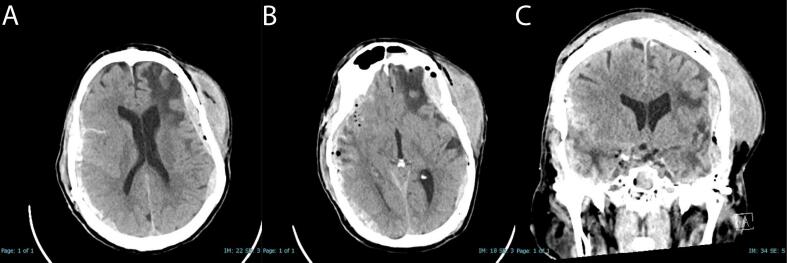
Initial admission non-contrast CT-scans - (A) Axial view highlighting the bilateral subdural hematoma component; (B) lower axial cut illustrating the bilateral epidural hematoma component. (C) Coronal view illustrates bilateral subdural hematoma.

**Fig. 2:**
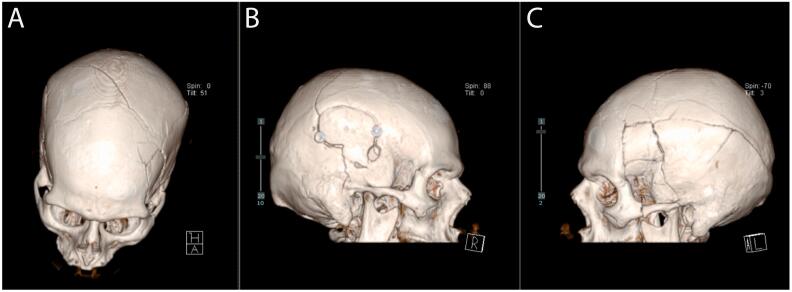
3D reconstruction of skull fractures. Image A shows a superior view of the fracture. Image B shows the right side of the skull, where the patient previously had a craniotomy. Image C displays the left side, where the new skull fractures occurred.

A non-contrast head CT scan performed within 1 h of admission revealed bilateral subdural hematomas with a potential epidural hematoma component, right frontal traumatic subarachnoid hemorrhage, and intraventricular hemorrhage (Fig. 1). There was a complex and mildly displaced calvarial fracture extending from the right parietal bone anteriorly to involve the left frontal and temporal bone. The complex fracture extended inferiorly to involve the petrous portion left temporal bone with underlying pneumocephalus (Fig. 2).

Given the grave nature of his prognosis, his family expressed preference towards comfort measures and organ donation. However, within 24 h of admission, he markedly improved to a GCS score of 9 T in the ICU. He was awake, able to follow some commands, and move all extremities spontaneously and purposefully. After discussion of all options with the family and IDN, the family decided to resume care and pursue transfer to our main facility. Routine coagulation studies revealed an international normalized ratio (INR) of 1.57, fibrinogen level of 60 mg/dL, thrombocytopenia with a platelet count of 103,000, raising concern for disseminated intravascular coagulation (DIC). Blood products and colloid were given to control coagulopathy and achieve adequate stabilization for transfer.

Upon transfer, the neurosurgical team deferred immediate surgical intervention due to the absence of significant mass effect or midline shift. Interventional radiology was consulted and for treatment options for the splenic injury and ultimately decided on conservative management with further imaging studies in one week. Orthopedic surgery was consulted for bilateral scapula and left distal clavicle fractures and recommended non operative management. Neurosurgical management included hypertonic saline and levetiracetam to manage [cerebral edema and mitigate the risk of seizures. Blood work showed worsening thrombocytopenia (platelet count 48,000), while thromboelastography (TEG), revealed decreased maximum] amplitude (MA) values for arachidonic acid (AA) (11.3 mm) and adenosine diphosphate (ADP) (12.5 mm). These findings prompted desmopressin (DDAVP) administration.

**Fig. 3:**
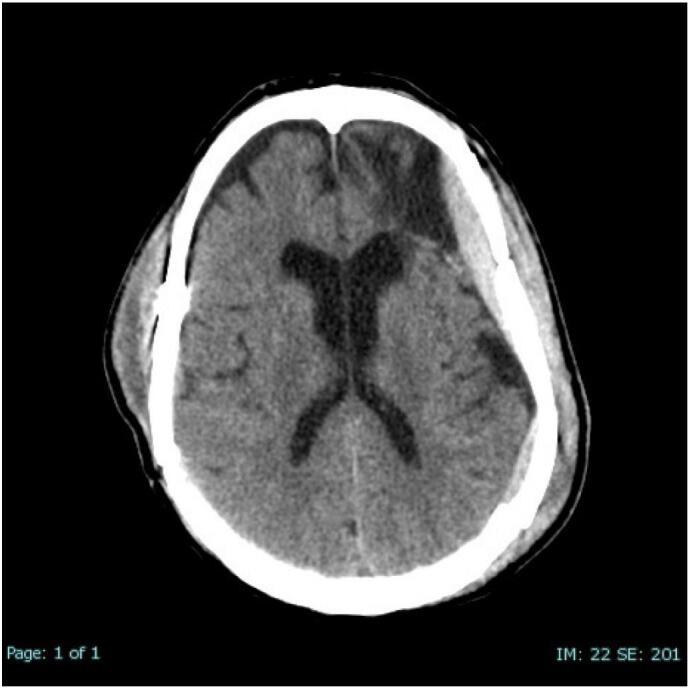
Follow-up CT scan - Demonstrating spontaneous resolution of bilateral subdural hematomas.

By 72 h of admission, platelet counts improved to 94,000 with transfusions. MA AA improved to 27 mm and MA ADP improved to 60.7 mm. A repeat non-contrast CT head showed significant improvement bilateral acute SDH with stable left temporal EDH and bilateral skull fractures (Fig. 3). On examination, he was evaluated to be GCS 10 T, moving all extremities vigorously and strongly when off sedation, and following commands. Given these findings, the neurosurgery team recommended close neurological checks, continuing keppra for 7 days, but no surgical intervention. Our team signed off and care was continued by the ICU team.

Unfortunately, the patient subsequently developed acute respiratory distress syndrome (ARDS) one week after the trauma. Despite aggressive interventions and support, his respiratory status deteriorated further, with the eventual onset of pneumonia. Palliative care was consulted, and goals of care were discussed with the family. They ultimately decided to make the patient DNR with comfort care. Donation after circulatory death (DCD) was decided by the family but was unable to be completed in the allotted time frame, resulting in the patient's death–one month after the fall.

## 4 Clinical discussion

The case exemplifies not only TBI, but the problems of the polytraumatized patient. This patient had TBI with intracranial bleeds, background seizure disorder, blunt chest trauma requiring a chest tube for hemothorax, a major splenic injury, spinal fractures and hemorrhagic shock with its attendant sequalae to other organs. We highlight the complex and unpredictable nature of TBI management and prognosis, reinforcing the critical importance of maintaining a cautiously optimistic approach in managing severe TBI. This aligns with the findings of Bonds et al. [6] and Dijkland et al. [10] who noted the challenges in accurately predicting TBI outcomes and the utility of prognostic models.

The decision to not initially operate on this patient's intracranial hemorrhages was multifactorial. Initial CT at the outside hospital (OSH) revealed extensive intracranial hemorrhages across multiple compartments, associated with complex calvaria fractures but without significant mass effect or midline shift, partially due to preexisting encephalomalacia. When neurosurgery was consulted, immediate surgical intervention was deferred given the absence of midline shift or mass effect. The patient's family initially decided on comfort measures for the patient and potential organ donation due to the patients declining clinical status most likely attributed to multi-organ failure. However, rapid clinical improvement prompted reconsideration and transfer to our main facility. Repeat neurosurgical evaluation again deferred surgery due to severe thrombocytopenia. With correction of platelet levels and ongoing neurological recovery, repeat imaging demonstrated resolution of hematomas without surgical intervention, favoring continued medical management.

Understanding the pathophysiology of TBI is critical for developing individualized treatment protocols. This case highlighted a distinctive instance of auto-decompression after a significant fall, necessitating a tailored approach responsive to abrupt clinical changes [9,15]. The patient exhibited an extensive, almost circumferential left-sided skull fracture, extending from anterior to posterior and inferior to superior directions involving the left frontal and temporal bones plus right parietal bone, akin to a large surgical craniotomy bone flap (Fig. 2). Research, such as that by Bramlett and Dietrich [16], reinforces the imperative of grasping the pathophysiological underpinnings within TBI to formulate effective management strategies. This complex fracture pattern likely facilitated the auto-decompression process by creating additional spaces for hematoma redistribution.

In analyzing the factors contributing to the resolution of hematomas and neurologic symptoms in our patient, it's crucial to consider various influences. The immediate improvement of GCS score could be attributed to resolution of a post-ictal state or improvement from hypovolemic shock. However, there was no massive internal or hemorrhage identified on FAST and CT imaging, other than the moderate hemothroax. There was no witness of seizure, however, due to this patient's history of seizure disorder and unknown medication adherence, it could be a possibility. Additionally, it is important to emphasize that patients did not have notable clinical features for a seizure. Still, there must be consideration that the patient's initial low GCS and neurological status reflected both multisystem trauma and hemorrhagic shock, with improvement following successful resuscitation.

This case, characterized by coagulopathies, chronic alcoholism, splenic injury, and brain atrophy, provides insight into how these conditions might facilitate the spontaneous resolution of hematomas. Firstly, the enlarged subdural and subarachnoid spaces could aid in the dispersal of the hematoma [13]. Secondly, Chaudhary et al. reported a case involving a 73-year-old male with myelodysplastic syndrome and chronic pancytopenia on warfarin therapy that had an unexpected resolution of an acute subdural hematoma (aSDH) [17]. His situation was further complicated by thrombocytopenia and significant warfarin-induced coagulopathy, which likely hindered stable blood clot formation. This deficit in coagulation is believed to have assisted in the dissolving and redistribution of the subdural blood, influenced by the movement of cerebral spinal fluid (CSF). This could have led to the spontaneous resolution of the hematoma. Coagulopathies, while shown to be associated with significantly worse outcomes and higher mortality rates, have continually been linked to rapid resolution of acute subdural hematomas [13,17,18]. Similarly, our patient's coagulopathy–evidenced by low fibrinogen, thrombocytopenia, and abnormal TEG findings–could have contributed to his hematoma resolution. Chronic alcoholism may have further influenced this patient's recovery. Recent literature has discussed rapid resolution of subdural hematomas in chronic alcoholics [19,20]. For instance, a 49-year-old female with a history of chronic alcoholism exhibited an unusual aSDH resolution following an assault. Found unconscious with a Glasgow Coma Score of 3 and a large left-sided aSDH causing significant midline shift, she remarkably recovered fully without surgical intervention [19]. While these cases report spontaneous resolution in patients with alcohol use disorder, these were often accompanied by anormal liver function, which were not prominent in this patient.

This case highlights the unpredictable nature of neurological recovery in severe TBI, both acutely and in the long-term [21]. It demonstrates the necessity of continuous neurological evaluation and the capacity to adapt to rapid shifts in the patient's condition. This approach ensures comprehensive patient care and readiness for unexpected recoveries in the complex landscape of TBI treatment.

## 5 Conclusion

This case demonstrates how preexisting skull defects, complex fracture patterns, and alcohol use disorder can potentially facilitate auto-decompression in TBI patients. The combination of prior craniotomy, extensive new skull fractures, and acute coagulopathy likely created favorable conditions for spontaneous hematoma resolution. Our patient's unique pathophysiology expands our understanding of factors that may predict spontaneous resolution in acute subdural hematomas, while reinforcing the importance of serial neurological assessments in polytrauma patients with TBI.

## Author contribution

Conception and Design: BOG, AC, JO, GM. Acquisition of Data: All Authors. Analysis and Interpretation of data: All authors. Manuscript Drafting: all authors. Critically revising the article: all authors. Review submitted version of manuscript: AC, JO, GM. Approved the final version of the manuscript on behalf of all authors: GM.

## Consent

Written informed consent was obtained from next of kin for publication of this case report and accompanying images. A copy of the written consent is available for review by the Editor-in-Chief of this journal on request.

## Ethical approval

This case report was deemed exempt from ethical approval by the Indiana University School of Medicine Institutional Review Board (IRB) as it describes the course and outcome of a single patient case, with all patient identifiers removed to ensure confidentiality. Written informed consent was obtained from the patient's next of kin for the publication of this case report and accompanying images.

## Guarantor

Dr. Gordon Mao.

## Research registration number

As this is a single retrospective case report and not a “First in Man” study or clinical trial, registration was not required according to the Declaration of Helsinki 2013 guidelines. This report describes standard-of-care treatment and spontaneous clinical findings rather than a novel intervention or experimental procedure.

## Funding

No funding.

## Conflict of interest statement

The authors have no personal, financial, or institutional interest in any of the drugs, materials, or devices described in this article.
